# Single-cell transcriptomics reveals cellular heterogeneity and neoadjuvant chemotherapy response signatures in triple-negative breast cancer

**DOI:** 10.3389/fonc.2026.1892582

**Published:** 2026-08-19

**Authors:** Menglong Gao, Zhen Liu, Lili Xi, Hongliang Duan, Jingjing Guo

**Affiliations:** 1 Faculty of Applied Sciences, Macao Polytechnic University, Macau SAR, China; 2 The Second Affiliated Hospital of Chengdu Medical College, Nuclear Industry 416 Hospital, Chengdu, China; 3 Medical Laboratory Center, The First Hospital of Lanzhou University, Lanzhou, China

**Keywords:** cellular heterogeneity, neoadjuvant chemotherapy response, single-cell RNA sequencing, triple-negative breast cancer, tumor microenvironment

## Abstract

**Background:**

Triple-negative breast cancer (TNBC) shows marked intratumoral heterogeneity and variable responses to neoadjuvant chemotherapy (NAC), but the cellular determinants of treatment response remain incompletely defined.

**Methods:**

We analyzed the scRNA-seq dataset GSE161529 to characterize TNBC cellular states and used bulk cohorts, including GSE25066 and GSE58812, for NAC response-related signature evaluation and clinical validation. Cell types and subtypes were identified by t-SNE clustering. A permutation-based NACR score estimated chemotherapy-response potential. Differentiation hierarchies were reconstructed using CytoTRACE 2 and Monocle 2, and cell-cell communication was inferred with CellChat. Prognostic value was evaluated by Kaplan-Meier analysis, drug sensitivity was predicted using pRRophetic/GDSC, and *in vitro* validation used MDA-MB-231-derived C2-like sensitive and C4-like resistant TNBC models.

**Results:**

Nine major cell populations were identified, with cancer cells (49.3%) showing the highest NACR scores. Four cancer cell subtypes were delineated: C2 was enriched in predicted NACR-high groups, whereas C3/C4 correlated with low predicted response. Three tumor-associated macrophage (TAM) subtypes were identified, with TAMs + SLPI associated with high predicted NACR and TAMs + CXCL9 with low predicted NACR. Pseudotime analysis revealed increasing NACR scores along the cancer cell trajectory (C4 to C2) and decreasing scores along the TAM trajectory. Predicted NACR-high tumors showed dominant CAF-cancer cell interactions, whereas predicted NACR-low tumors showed endothelial cell-CAF/TAM communication via PDGFB-PDGFRB and ICAM2-ITGAM/ITGB2. The NACR signature was associated with overall survival (log-rank p = 0.034). *In vitro* experiments confirmed a 12.81-fold IC50 difference between C2-like and C4-like models and validated subtype markers.

**Conclusions:**

This integrative analysis identifies response-associated cancer cell and TAM states and nominates PDGFB-PDGFRB signaling as a candidate resistance-associated pathway, providing potential biomarkers and therapeutic targets for precision TNBC therapy.

## 1 Introduction

Triple-negative breast cancer (TNBC), defined by the absence of estrogen receptor, progesterone receptor, and HER2 overexpression, accounts for 15–20% of breast cancer cases and is characterized by aggressive clinical behavior, high relapse rates, and limited targeted therapeutic options (1–3). Neoadjuvant chemotherapy (NAC) remains the standard-of-care treatment for locally advanced TNBC, offering opportunities for tumor downsizing and surgical optimization. However, approximately 30–40% of patients fail to achieve pathological complete response (pCR), and the molecular mechanisms underlying this heterogeneous treatment response remain poorly understood (4, 5).

The tumor microenvironment (TME) of TNBC is highly complex, comprising cancer cells, immune cells, stromal cells, and endothelial cells, whose dynamic interactions significantly influence treatment response and clinical outcomes (6, 7). Single-cell RNA sequencing (scRNA-seq) has emerged as a transformative tool for dissecting cellular heterogeneity and intercellular communication within the TME at unprecedented resolution (8, 9). While previous bulk transcriptomic studies have provided valuable insights into TNBC molecular subtypes, they lack the power to resolve rare cell populations, characterize cell-type-specific responses to NAC, or capture the dynamic interplay among TME components (10, 11).

**Figure 1:**
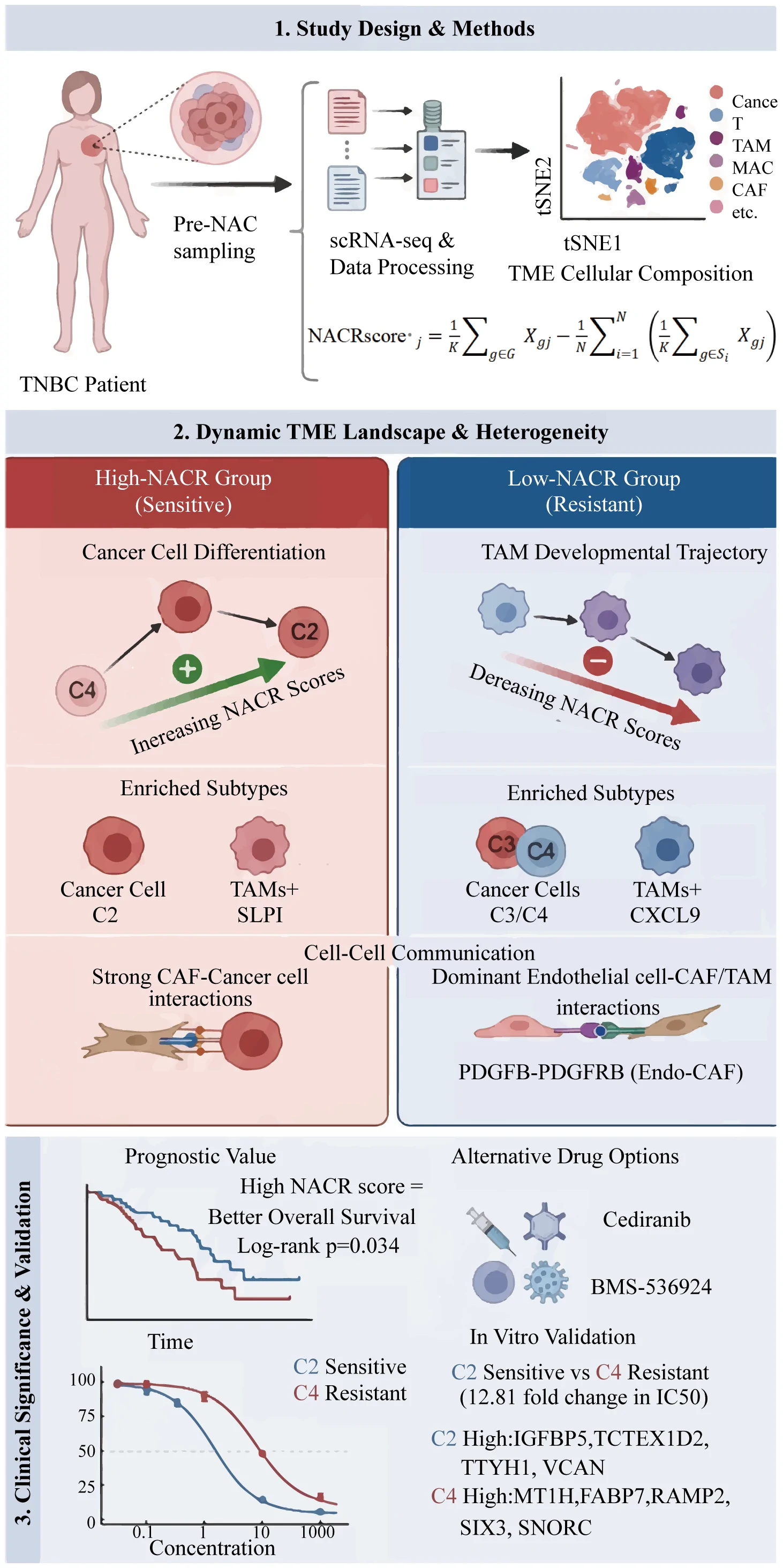
Workflow.

Here, we employed scRNA-seq to profile TNBC samples before NAC, with the following objectives: 1) to characterize the cellular composition of the TME; 2) to identify cancer cell and immune cell subtypes associated with predicted NAC response; 3) to reconstruct developmental trajectories of cancer cells and tumor-associated macrophages (TAMs); 4) to map cell-cell communication networks associated with predicted NAC response states; and 5) to validate the clinical relevance of the identified signatures through survival analysis, drug sensitivity prediction, and *in vitro* experimental verification. We developed a neoadjuvant chemotherapy response (NACR) scoring system based on established markers of chemotherapy sensitivity and resistance, enabling quantification of predicted NAC response potential at the single-cell level and linking it to cellular heterogeneity and TME dynamics. By integrating computational analyses with experimental validation, this study aims to provide a framework for understanding how TME components may contribute to heterogeneous NAC responses and to identify candidate biomarkers and therapeutic targets for precision medicine in TNBC (**Figure 1**).

## 2 Materials and methods

### 2.1 Data processing

The scRNA-seq dataset used for single-cell analysis was obtained from the Gene Expression Omnibus (GEO) database under accession GSE161529 (12), and bulk transcriptomic datasets (GSE25066 and GSE58812) were used for NAC response-related signature evaluation and clinical validation. The NACR (neoadjuvant chemotherapy response) gene set was derived from published literature and was applied consistently across single-cell and bulk expression matrices after normalization. The scRNA-seq data underwent the following preprocessing steps:

#### 2.1.1 Quality control

Cells were filtered based on the criteria: number of genes per cell ranged between 500 and 6000, UMI counts within 1000–10000, and mitochondrial UMI proportion ≤10% (13).

#### 2.1.2 Data integration and normalization

The Seurat SCTransform method was applied for normalization (14). The top 2000 highly variable genes and variance-stabilizing genes were selected for principal component analysis (PCA).

#### 2.1.3 Cell annotation

t-SNE plots were constructed using principal components, and cell types were identified by key marker genes. Subtypes were classified based on cluster-specific highly expressed genes (15).

### 2.2 Single-cell-based NACR state evaluation

To quantify the predicted neoadjuvant chemotherapy response (NACR) potential at single-cell resolution, we constructed a literature-derived NACR gene set containing genes previously associated with chemotherapy sensitivity or resistance in TNBC and breast cancer. Genes reported as response-favorable were treated as positive NACR genes, whereas genes reported as resistance-associated were treated as negative NACR genes.

For each cell, normalized SCTransform expression values were used to calculate a NACR score. Briefly, the mean expression of positive NACR genes was subtracted by the mean expression of negative NACR genes and then adjusted against randomly sampled control genes with matched expression levels. Random control gene sets of the same size were sampled repeatedly to generate a null distribution, and the final NACR score was defined as the observed signature score minus the mean control score across permutations. Higher NACR scores therefore indicate a higher predicted chemotherapy-response potential.

Cells or bulk samples were stratified into NACR-high and NACR-low groups according to the median NACR score unless otherwise specified. This classification represents a transcriptomic prediction of response-associated state rather than direct pathological complete response for samples without clinical NAC outcome information. The same scoring direction and cutoff strategy were used in downstream single-cell, survival, and drug-sensitivity analyses to maintain consistency across datasets.

The NACR gene list used for scoring is provided in **Supplementary Table 1**.

### 2.3 Cell type distribution preferences

To describe cell type distribution preferences across NACR score-defined groups, odds ratios (ORs) were calculated. For each combination of cell type i and category j, a 2 x 2 contingency table was formed. Fisher’s exact test was used to calculate OR values and corresponding p-values. The p-values were adjusted using the Benjamini-Hochberg (BH) method (16) implemented in the R function p.adjust. A larger OR value indicates that cell type i is more likely to appear in category j, while a smaller OR value indicates reduced enrichment in that NACR score-defined group.

### 2.4 Trajectory and differentiation state analysis

Cell differentiation potential was first assessed using CytoTRACE 2, a computational framework that predicts the relative differentiation status of individual cells from scRNA-seq data based on transcriptional diversity (17). CytoTRACE 2 scores range from more differentiated to less differentiated, providing an independent, unsupervised estimate of cellular maturity. CytoTRACE 2 analysis was applied to both cancer cell subtypes and tumor-associated macrophage (TAM) subtypes to establish their relative differentiation hierarchy prior to pseudotime ordering.

Subsequently, pseudotime trajectories were reconstructed using Monocle 2 (18). Differentially expressed genes across subtypes were identified by the differential GeneTest function (Benjamini–Hochberg-corrected p < 0.01), and cells were ordered along pseudotime in an unsupervised manner using the DDRTree dimensionality reduction algorithm with default parameters. The inferred differentiation direction was annotated by arrows based on the concordance between CytoTRACE 2 differentiation scores and pseudotime ordering: cells with lower differentiation status (less differentiated, as indicated by CytoTRACE 2) were assigned to the root of the trajectory. Heatmaps were generated using ComplexHeatmap to visualize cell type composition and marker gene expression dynamics along pseudotime (19).

### 2.5 Cellular communication

Cell-cell communication networks were constructed using the CellChat R package (20). Ligand-receptor expression averages within interacting clusters were calculated via 1000 random permutations of cluster labels to generate null distributions. p-values for receptor-ligand pairs were determined by comparing observed means to null distributions, and significant interactions were filtered. Communication differences between predicted NACR-high and NACR-low groups were visualized via heatmaps, and specific signaling pathways were analyzed for outward and inward signaling patterns.

### 2.6 Survival analysis and drug sensitivity prediction

Overall survival (OS) analysis was evaluated using the public TNBC dataset GSE58812 with associated clinical information. Kaplan-Meier survival curves were generated to compare OS between NACR score-defined predicted NACR-high and predicted NACR-low groups, and differences were assessed using the log-rank test. The NACR signature was interpreted as being associated with survival rather than as an independent prognostic factor unless multivariate Cox regression was performed. To identify potential therapeutic agents, the pRRophetic R package was utilized to predict the half-maximal inhibitory concentration (IC50) of multiple small-molecule drugs based on the Genomics of Drug Sensitivity in Cancer (GDSC) database (21, 22). The estimated IC50 values were compared between the NACR score-defined predicted NACR-high and predicted NACR-low groups using the Wilcoxon rank-sum test.

### 2.7 Cell viability assay

The cell lines present in this study were obtained from the Shanghai Institutes for Biological Sciences, specifically the Cell Resource Center of the Shanghai Institute for Biological Sciences, Chinese Academy of Sciences, located in Shanghai, China.

To validate the differential chemotherapy sensitivity between C2-like and C4-like subtypes, a drug-induced resistant MDA-MB-231-derived condition was established from parental MDA-MB-231 cells. The parental cells were maintained as the drug-sensitive comparator. The C2-like and C4-like designations refer to the parental sensitive and drug-induced resistant experimental conditions, respectively. Cell viability was measured using the Cell Counting Kit-8 (CCK-8) assay. Briefly, cells were seeded in 96-well plates, allowed to adhere, and treated with serial concentrations of the chemotherapeutic drug for a defined incubation period. CCK-8 reagent was then added, and absorbance was measured at 450 nm using a microplate reader. Dose-response curves were fitted using a nonlinear regression model, and IC50 values were calculated to determine drug sensitivity. CCK-8 experiments were performed with six biological replicates (n = 6) for each group.

### 2.8 Real-time quantitative PCR and immunofluorescence

Total RNA was extracted from MDA-MB-231-derived C2-like sensitive and C4-like resistant cells using TRIzol reagent and reverse-transcribed into cDNA. RT-qPCR was performed with six biological replicates (n = 6) per group to detect the relative mRNA expression levels of key marker genes (MT1H, FABP7, RAMP2, SIX3, SNORC, IGFBP5, TCTEX1D2, TTYH1, and VCAN), normalized to GAPDH and calculated using the 2^-ΔΔCt method. Primer sequences are listed in **Supplementary Table 2**. For immunofluorescence (IF) staining, cells were fixed, permeabilized, and blocked, followed by overnight incubation with primary antibodies against FABP7 and IGFBP5. Cells were then incubated with fluorophore-conjugated secondary antibodies, and nuclei were counterstained with DAPI. Images were captured using a fluorescence microscope, and the mean fluorescence intensity (MFI) was quantified.

### 2.9 Statistical analysis

Statistical analyses were performed using R (version 4.3.1). Parametric tests (Student’s t-test, ANOVA) were used for normally distributed data, and non-parametric tests (Wilcoxon, Kruskal-Wallis) for non-normal data. Pearson’s/Spearman’s correlation was used to assess variable relationships, ensuring appropriate statistical validation of results.

## 3 Results

### 3.1 Single-cell clustering and cell type identification in triple-negative breast cancer patients

**Figure 2:**
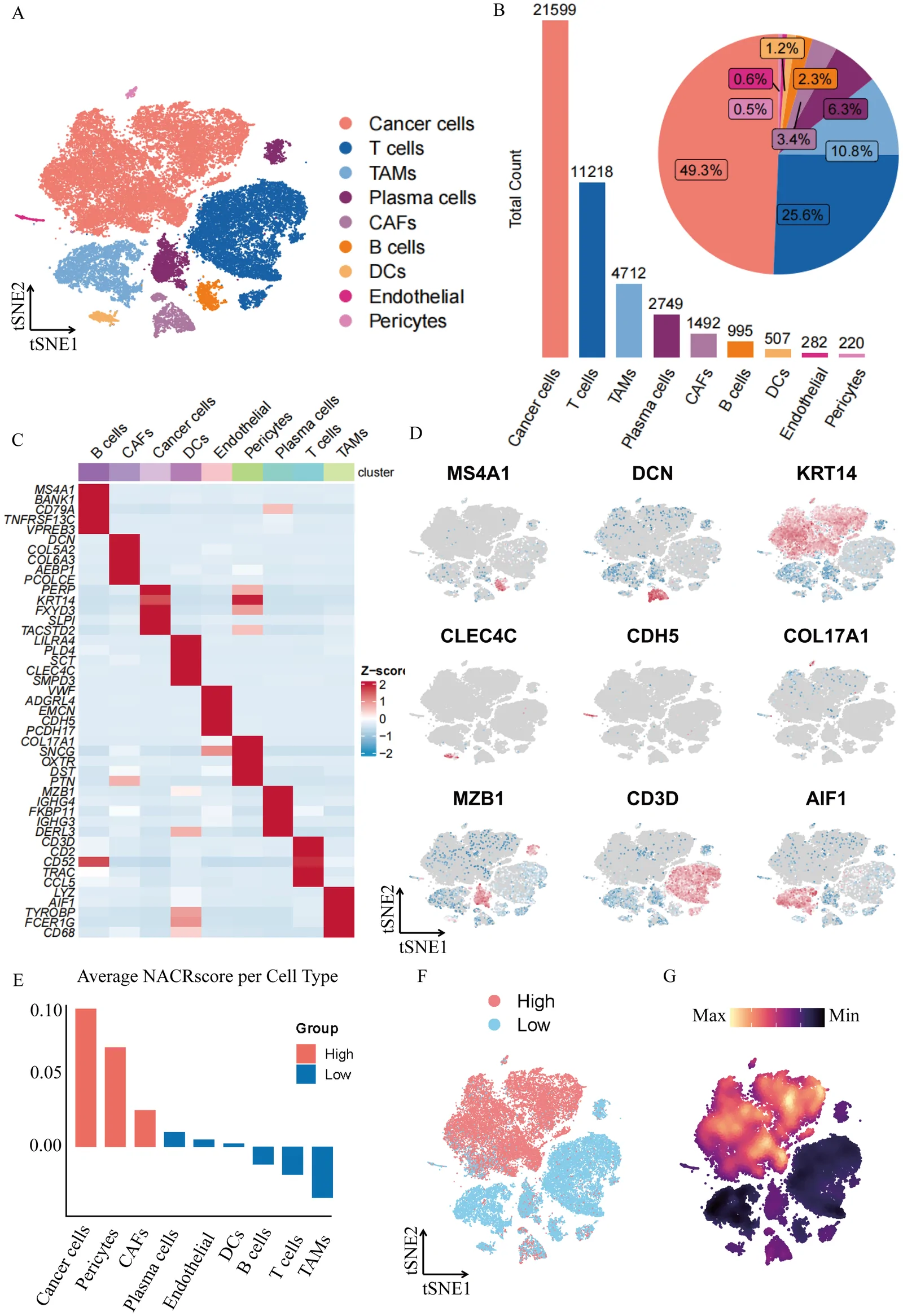
Single-cell clustering and characterization of the tumor microenvironment in triple-negative breast cancer (TNBC). **(A)** t-SNE plot depicting the major cell populations identified in TNBC, including cancer cells, T cells, tumor-associated macrophages (TAMs), plasma cells, cancer-associated fibroblasts (CAFs), B cells, dendritic cells (DCs), endothelial cells, and pericytes. **(B)** Bar chart showing the relative abundance (percentages) of each identified cell type in the tumor microenvironment. **(C)** Heatmap illustrating the expression of the top differentially expressed marker genes across the identified cell clusters. **(D)** t-SNE plots displaying the expression levels of representative cell-type-specific marker genes, such as MS4A1 (B cells), DCN (CAFs), and KRT14 (cancer cells). **(E)** Boxplot comparing the neoadjuvant chemotherapy response (NACR) scores across different cell types, with cancer cells exhibiting the highest scores. **(F)** t-SNE plot colored by patient groups with high and low NACR scores, illustrating spatial distribution differences. **(G)** Heatmap representation of NACR scores across cell clusters, highlighting single-cell heterogeneity in chemotherapy response.

Single-cell RNA-sequencing analysis was performed to characterize the cellular composition of the tumor microenvironment and neoadjuvant chemotherapy response (NACR)-related features in triple-negative breast cancer. t-SNE clustering (**Figure 2A**) identified major cell populations, including cancer cells, T cells, tumor-associated macrophages (TAMs), plasma cells, cancer-associated fibroblasts (CAFs), B cells, dendritic cells (DCs), endothelial cells, and pericytes, unveiling inherent cellular heterogeneity. Quantification of cell abundances (**Figure 2B**) showed cancer cells were the most prevalent (49.3% of total cells), followed by T cells (25.6%), TAMs (10.8%), plasma cells (6.3%), CAFs (3.4%), B cells (2.3%), DCs (1.2%), endothelial cells (0.6%), and pericytes (0.5%), highlighting the dominance of cancer cells and substantial immune/stromal components. A gene expression heatmap (**Figure 2C**) delineated cell-type-specific transcriptional signatures, with marker genes enriched in respective clusters, providing a molecular basis for cell type identification. Visualization of single-gene expressions (**Figure 2D**), such as MS4A1 (B cell marker), DCN (CAF-associated), and KRT14 (cancer cell-related), validated cell-type-specific patterns. NACR score analysis (**Figures 2E–G**) revealed cancer cells had the highest average NACR scores, with significant differences between predicted NACR-high and predicted NACR-low groups. t-SNE distribution (**Figure 2F**) and heatmap representation (**Figure 2G**) showed spatial separation and cluster-specific variation of NACR scores, indicating single-cell heterogeneity in chemotherapy response linked to microenvironmental composition.

### 3.2 Intratumoral heterogeneity of cancer cells in triple-negative breast cancer

**Figure 3:**
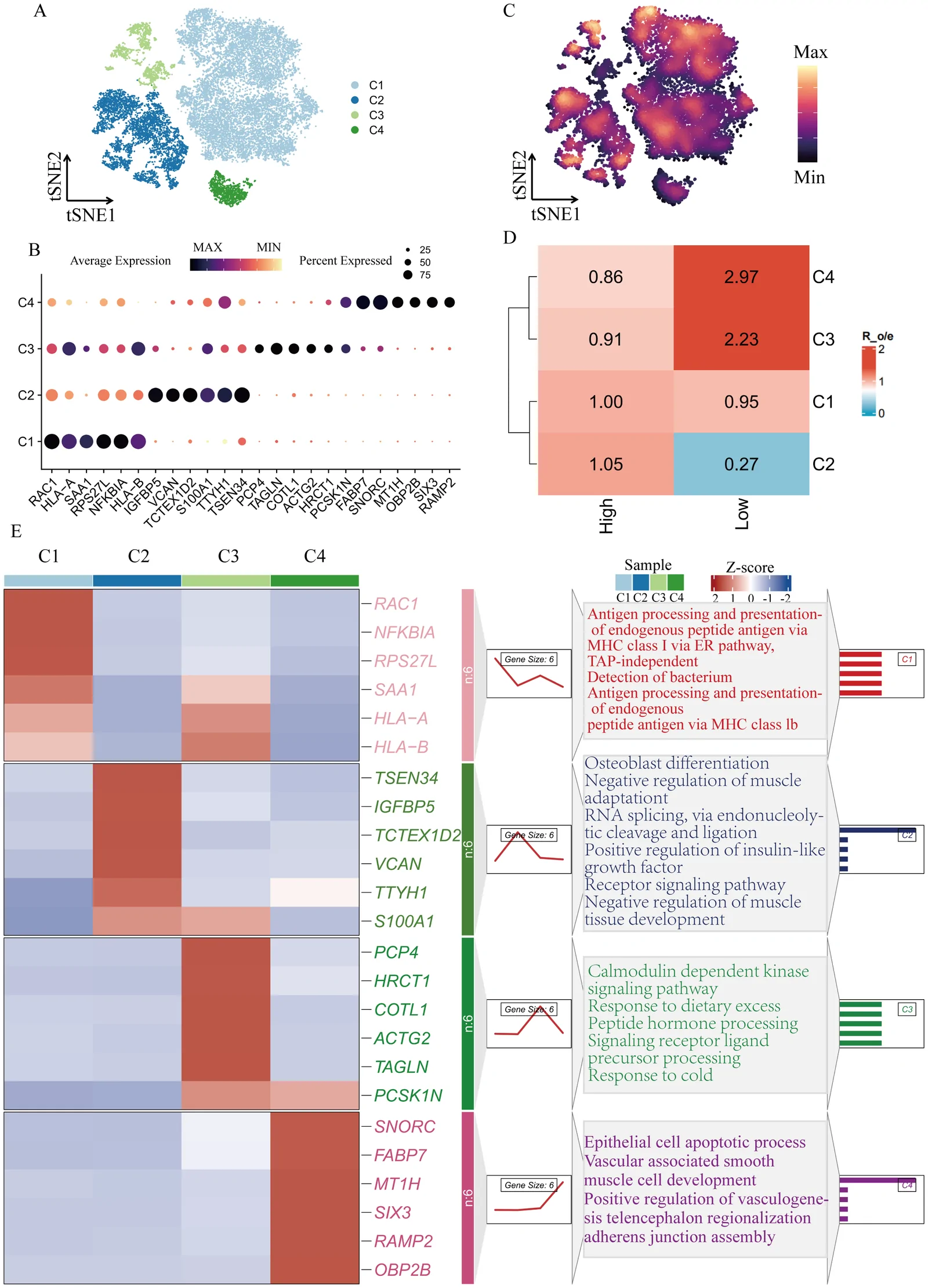
Intratumoral heterogeneity and molecular signatures of TNBC cancer cells. **(A)** t-SNE plot showing the sub-clustering of cancer cells into four distinct subtypes (C1, C2, C3, and C4). **(B)** Bubble plot visualizing the expression patterns of subtype-specific differential genes across the C1–C4 subpopulations. **(C)** Distribution of NACR scores among the four cancer cell subtypes. **(D)** Odds ratio (OR) analysis evaluating the distribution preferences of C1–C4 subtypes in predicted NACR-high versus predicted NACR-low groups. **(E)** Heatmap and corresponding functional enrichment analysis of the top six differentially expressed genes for each cancer cell subtype, linking them to specific biological pathways (e.g., antigen processing for C1, apoptosis for C4).

Sub-clustering of cancer cells was performed to explore intratumoral heterogeneity. t-SNE analysis (**Figure 3A**) partitioned cancer cells into four subtypes (C1, C2, C3, C4), revealing diverse subpopulations. A bubble plot (**Figure 3B**) visualized differential gene expression patterns, highlighting subtype-specific molecular features. NACR score distribution across subtypes (**Figure 3C**) showed distinct responses to neoadjuvant chemotherapy. OR value analysis (**Figure 3D**) indicated C2 was more associated with the NACR predicted NACR-high group, whereas C3 and C4 had lower odds of high response. A heatmap of the top six differentially expressed genes per subtype (**Figure 3E**) with enrichment analysis revealed that C1-C4 were linked to pathways such as antigen processing, osteoblast differentiation, signaling, and apoptosis, respectively. These findings demonstrate that cancer cell subtypes exhibit distinct molecular signatures and clinical implications, contributing to heterogeneous therapeutic responses.

### 3.3 Heterogeneity and functional characterization of tumor-associated macrophages

**Figure 4:**
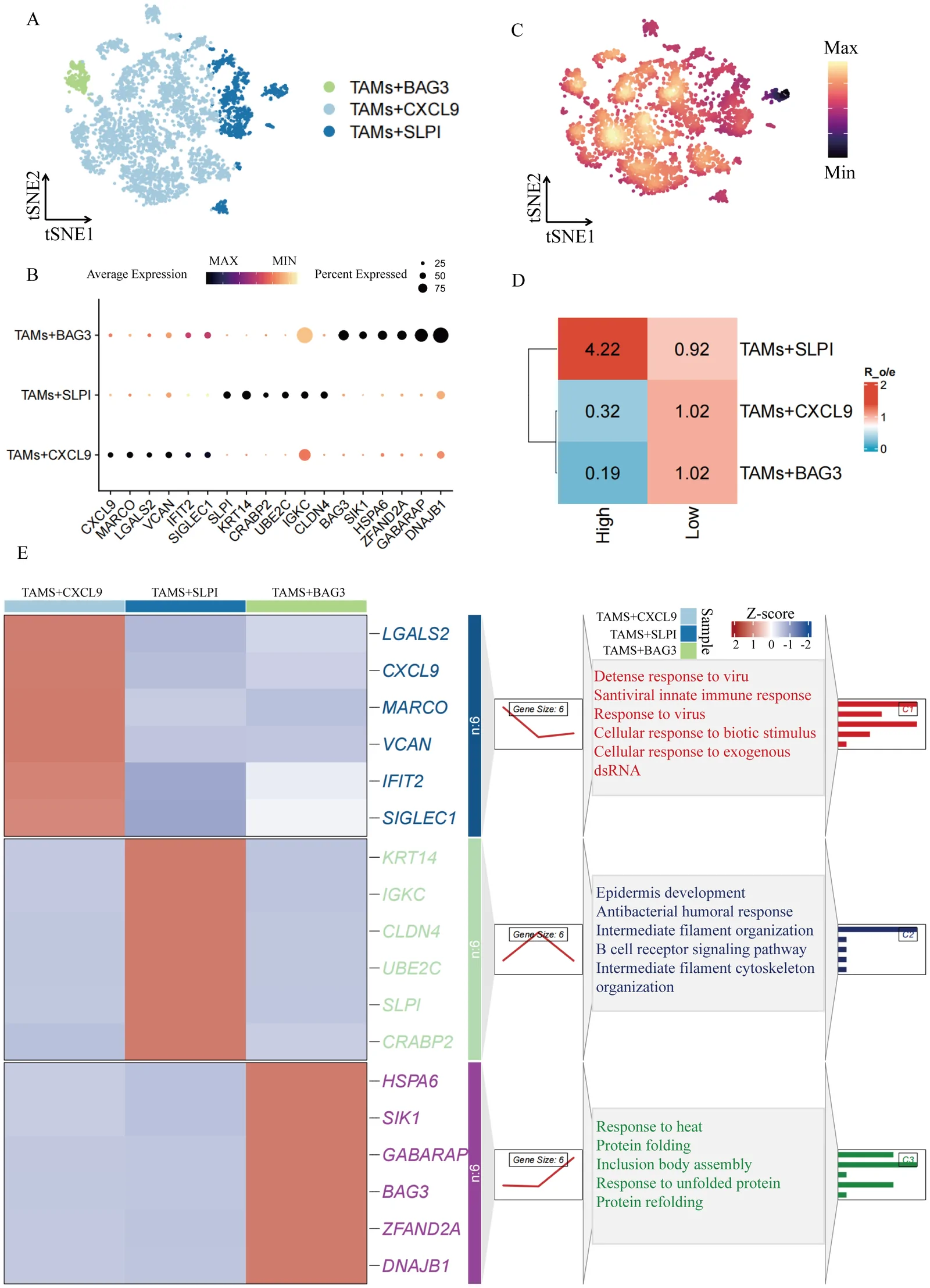
Heterogeneity and functional states of tumor-associated macrophages (TAMs). **(A)** t-SNE plot displaying the sub-clustering of TAMs into three functional sub-populations: TAMs + BAG3, TAMs + CXCL9, and TAMs + SLPI. **(B)** Bubble plot showing the expression of signature genes defining the three TAM subtypes. **(C)** Distribution of NACR scores across the different TAM subtypes. **(D)** Odds ratio (OR) analysis for the preferential enrichment of TAM subtypes in the predicted NACR-high versus predicted NACR-low groups. **(E)** Pathway enrichment analysis indicating the distinct biological functions associated with each TAM subtype (e.g., TAMs + CXCL9 in antiviral defense, TAMs + SLPI in antibacterial response, and TAMs + BAG3 in heat response/protein folding).

Sub-clustering of TAMs was conducted to dissect their heterogeneity. t-SNE clustering (**Figure 4A**) segregated TAMs into three subtypes: TAMs + BAG3, TAMs + CXCL9, and TAMs + SLPI, indicating functional diversity. A bubble plot (**Figure 4B**) visualized differential gene expression, highlighting subtype-specific molecular signatures. NACR score distribution (**Figure 4C**) suggested distinct roles in chemotherapy response modulation. OR analysis (**Figure 4D**) showed TAMs + SLPI was associated with the predicted NACR-high group, while TAMs + CXCL9 was linked to low response. Pathway enrichment analysis (**Figure 4E**) revealed TAMs + CXCL9 was involved in antiviral defense, TAMs + SLPI in epidermal development/antibacterial response, and TAMs + BAG3 in heat response/protein folding, indicating diverse functional implications for microenvironmental heterogeneity and chemotherapy response.

### 3.4 Developmental trajectories of cancer cell subtypes and NACR dynamics

**Figure 5:**
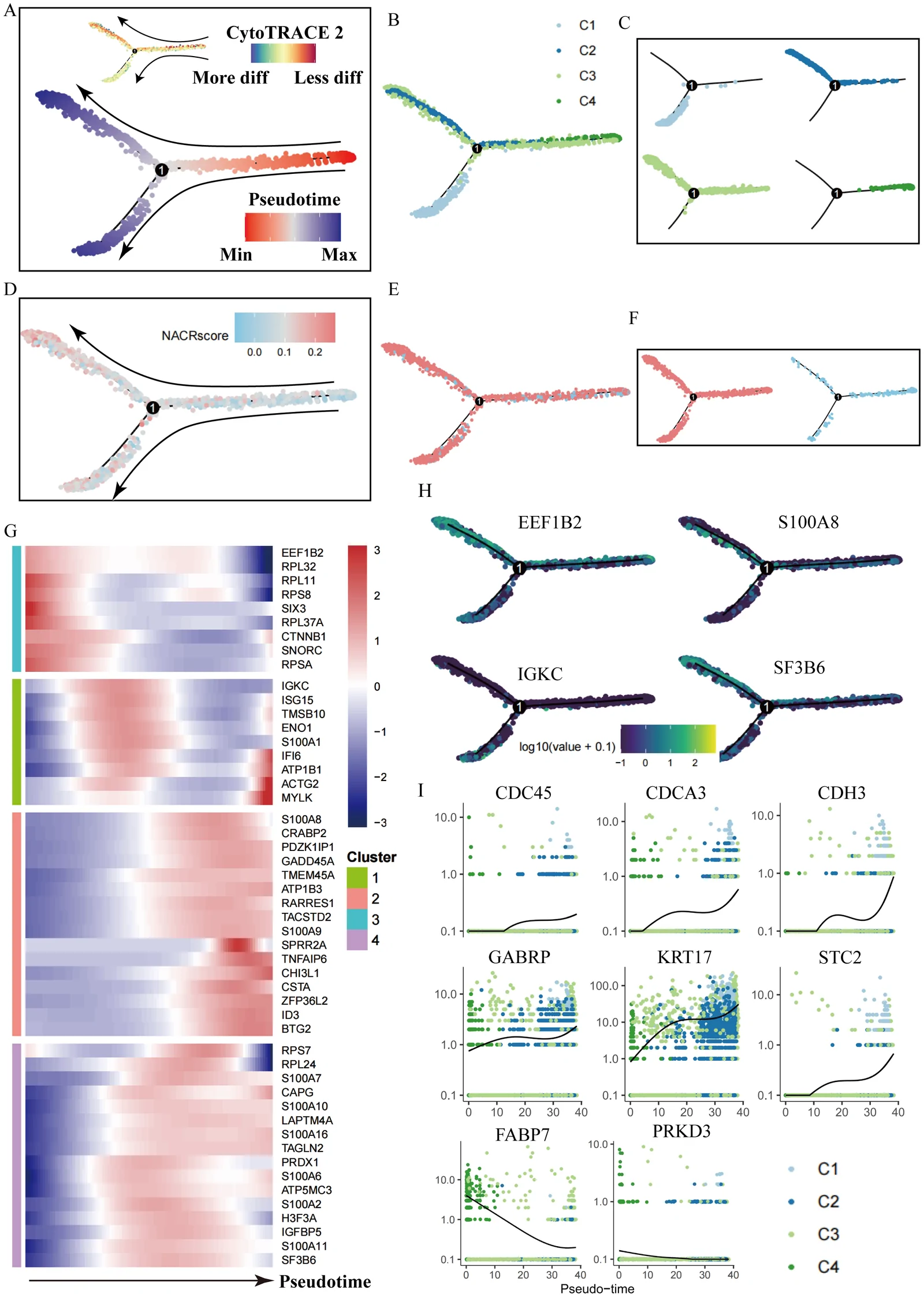
Pseudotime developmental trajectories of cancer cell subtypes and their association with NACR dynamics. **(A-C)** Pseudotime trajectory maps reconstructing the developmental paths and transitions among the C1–C4 cancer cell subtypes. **(D)** Scatter plot illustrating the progressive increase of NACR scores along the pseudotime trajectory, corresponding to the sequence from C4 to C3, C1, and C2. **(E)** Distribution of cells from predicted NACR-high and predicted NACR-low groups along the pseudotime trajectory. **(F, G)** Unsupervised clustering and visualization of cell state complexity along the trajectory. **(H)** Heatmap displaying the dynamic changes in cluster-specific gene expression along pseudotime. **(I)** Expression trends of specific NACR-related genes over pseudotime, showing distinct increasing or decreasing patterns that mirror the overall NACR trends.

To delineate the developmental hierarchy of cancer cell subtypes, we first applied CytoTRACE 2 to evaluate the relative differentiation status of each cell. CytoTRACE 2 analysis (**Figure 5A**, upper inset) revealed that C4 cells exhibited the least differentiated state, whereas C2 cells were the most differentiated, suggesting a differentiation gradient from C4 toward C2. Building on this differentiation hierarchy, we performed pseudotime trajectory reconstruction using Monocle 2 (**Figure 5A**, main panel). The inferred pseudotime ordering was consistent with the CytoTRACE 2 predictions: cells progressed from a less differentiated state (low pseudotime, corresponding to C4) toward a more differentiated state (high pseudotime, corresponding to C2), with C3 and C1 positioned at intermediate stages. Overlay of cancer cell subtypes on the trajectory (**Figure 5B**) confirmed the spatial segregation of C1-C4 along the pseudotime axis, and individual subtype trajectories (**Figure 5C**) further illustrated their distinct positions within the differentiation continuum.

Notably, NACR scores (**Figure 5D**) increased progressively along pseudotime, corresponding to the developmental sequence from C4 to C3, C1, and C2, indicating that cells at more advanced differentiation stages (such as C2) possessed higher neoadjuvant chemotherapy response potential. The predicted NACR-high and predicted NACR-low group distribution (**Figure 5E**) showed that high-response cells predominated in late pseudotime stages, and the distribution pattern across trajectory branches (**Figure 5F**) further corroborated this trend. Unsupervised clustering along the trajectory (**Figure 5G**) and gene expression heatmaps (**Figure 5H**) revealed cluster-specific transcriptional dynamics during differentiation. NACR-related genes (**Figure 5I**) showed increasing expression for the first six genes and decreasing expression for the last two along pseudotime, mirroring the NACR score trends and indicating dynamic gene regulation underlying chemotherapy response heterogeneity.

### 3.5 Developmental dynamics of TAM subtypes and NACR associations

**Figure 6:**
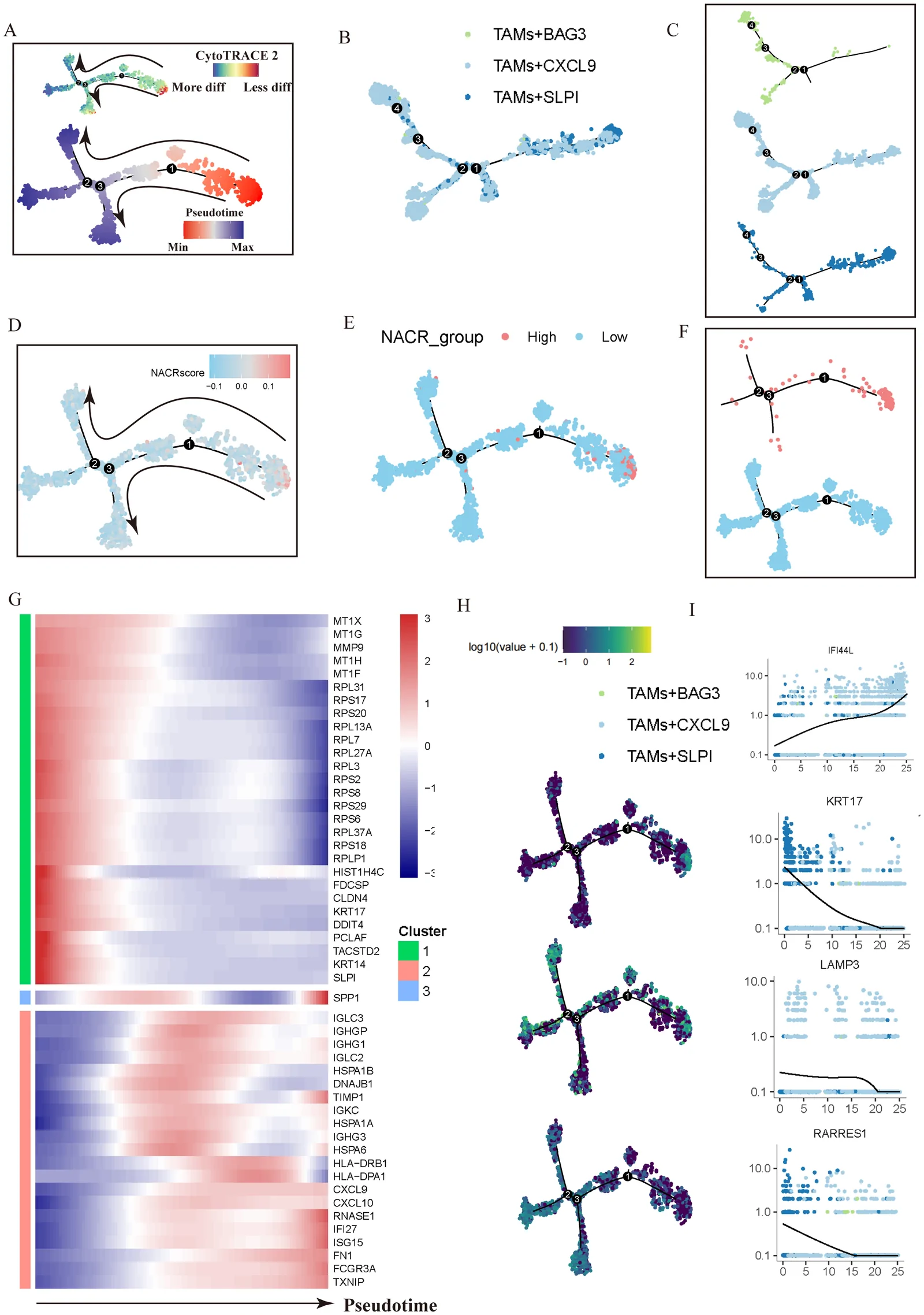
Developmental dynamics of TAM subtypes and their correlation with NACR scores. **(A-C)** Pseudotime trajectory plots delineating the developmental progression of the three TAM subtypes. **(D)** Scatter plot showing the continuous decrease in NACR scores along the TAM pseudotime trajectory. **(E, F)** Distribution of TAMs from predicted NACR-high and predicted NACR-low groups mapped onto the pseudotime trajectory. **(G)** Unsupervised clustering of TAMs along pseudotime. **(H)** Heatmap illustrating the dynamic transcriptional changes of cluster-specific genes along the developmental trajectory. **(I)** Dynamic expression patterns of selected NACR-related genes across the TAM pseudotime, reflecting the molecular modulation of chemotherapy response by distinct TAM states.

To investigate the developmental hierarchy of tumor-associated macrophage (TAM) subtypes, CytoTRACE 2 was first applied to assess their relative differentiation status. CytoTRACE 2 analysis (**Figure 6A**, upper inset) revealed that TAMs + BAG3 exhibited a less differentiated phenotype, while TAMs + SLPI displayed a more differentiated state, with TAMs + CXCL9 positioned at an intermediate stage. Guided by this differentiation hierarchy, Monocle 2 was employed to reconstruct pseudotime trajectories (**Figure 6A**, main panel). The trajectory exhibited a branching architecture with multiple branch points (nodes 1–3), reflecting the complex differentiation dynamics of TAMs. The pseudotime ordering was concordant with CytoTRACE 2 predictions, with cells progressing from a less differentiated origin toward distinct terminal states. Overlay of TAM subtypes on the trajectory (**Figure 6B**) demonstrated the spatial distribution of TAMs + BAG3, TAMs + CXCL9, and TAMs + SLPI along the pseudotime axis, and individual subtype trajectories (**Figure 6C**) further delineated their branching differentiation paths.

NACR scores (**Figure 6D**) decreased continuously along pseudotime, suggesting that TAMs at later developmental stages contributed less to favorable chemotherapy response. The predicted NACR-high and predicted NACR-low group distribution (**Figure 6E**) showed that high-response TAMs were concentrated in early pseudotime stages, and the spatial distribution across trajectory branches (**Figure 6F**) confirmed this pattern. Unsupervised clustering along the trajectory (**Figure 6G**) and gene expression dynamics (**Figure 6H**) revealed cluster-specific transcriptional patterns. NACR-related genes (**Figure 6I**) showed one gene with increasing expression and three genes with decreasing expression along pseudotime, aligning with NACR score trends and indicating molecular mechanisms of chemotherapy response modulation by TAM subtypes.

### 3.6 Intercellular communication patterns associated with NACR

**Figure 7:**
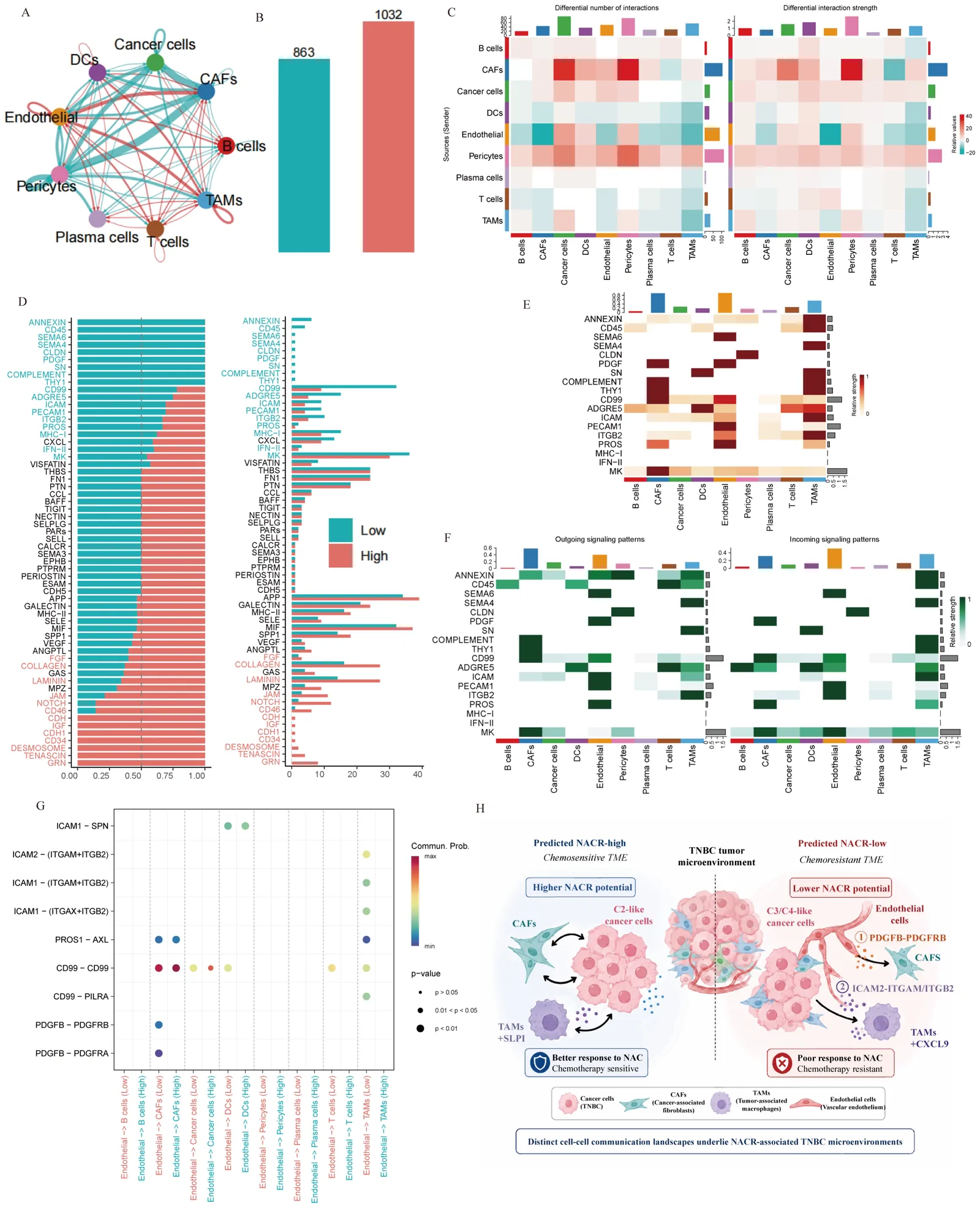
Intercellular communication networks underpinning differential NACR states. **(A)** Overview of the global cell-cell communication networks in the predicted NACR-high (red) and predicted NACR-low (blue) groups. **(B)** Bar chart quantifying the number of interactions within the predicted NACR-high and predicted NACR-low groups, demonstrating more active communication in the predicted NACR-high group. **(C)** Heatmaps comparing specific interaction strengths, highlighting CAF–cancer cell communication in the predicted NACR-high group versus endothelial cell–CAF/TAM interactions in the predicted NACR-low group. **(D, E)** Analysis of significant signaling pathway differences and receptor-ligand pairs between the response groups. **(F)** Bubble plots summarizing the signal emission and reception capacities of different cell types. **(G)** Specific analysis of key pathways enriched in the predicted NACR-low group, including PDGFB-PDGFRB (in endothelial cell–CAF interactions) and ICAM2-ITGAM/ITGB2 (in endothelial cell–TAM interactions). **(H)** Distinct cell-cell communication landscapes underlie NACR-associated TNBC microenvironments.

Cell-cell communication networks were analyzed to explore NACR-related interactions. The overall network (**Figure 7A**) showed predicted NACR-high and NACR-low groups, with the predicted NACR-high group exhibiting more interactions (**Figure 7B**), indicating active communication. Heatmaps (**Figure 7C**) showed the strongest CAF-cancer cell communication in the predicted NACR-high group, whereas endothelial cell-CAF and endothelial cell-TAM interactions dominated the predicted NACR-low group. Significant pathway interaction differences (**Figure 7D**), receptor-ligand summaries (**Figure 7E**), and signal emission and reception analyses (**Figure 7F**) highlighted candidate interactions, such as PDGFB-PDGFRB in endothelial cell-CAF communication and ICAM2-ITGAM/ITGB2 in endothelial cell-TAM communication (**Figure 7G**) in the predicted NACR-low group. These findings suggest distinct communication landscapes associated with predicted NACR states via CAFs, endothelial cells, and TAMs. **Figure 7H** provides a schematic overview of the NACR-associated communication model in TNBC. In the predicted NACR-high group, enhanced CAF-cancer cell crosstalk, together with the enrichment of C2-like cancer cells and TAMs + SLPI, may contribute to a microenvironment more permissive to chemotherapy response. In contrast, in the predicted NACR-low group, endothelial cell-driven communication with CAFs and TAMs, mediated in part by PDGFB-PDGFRB and ICAM2-ITGAM/ITGB2 signaling, may promote a stromal-immune niche associated with chemoresistance.

### 3.7 Prognostic value of NACR and screening of potential therapeutic drugs

**Figure 8:**
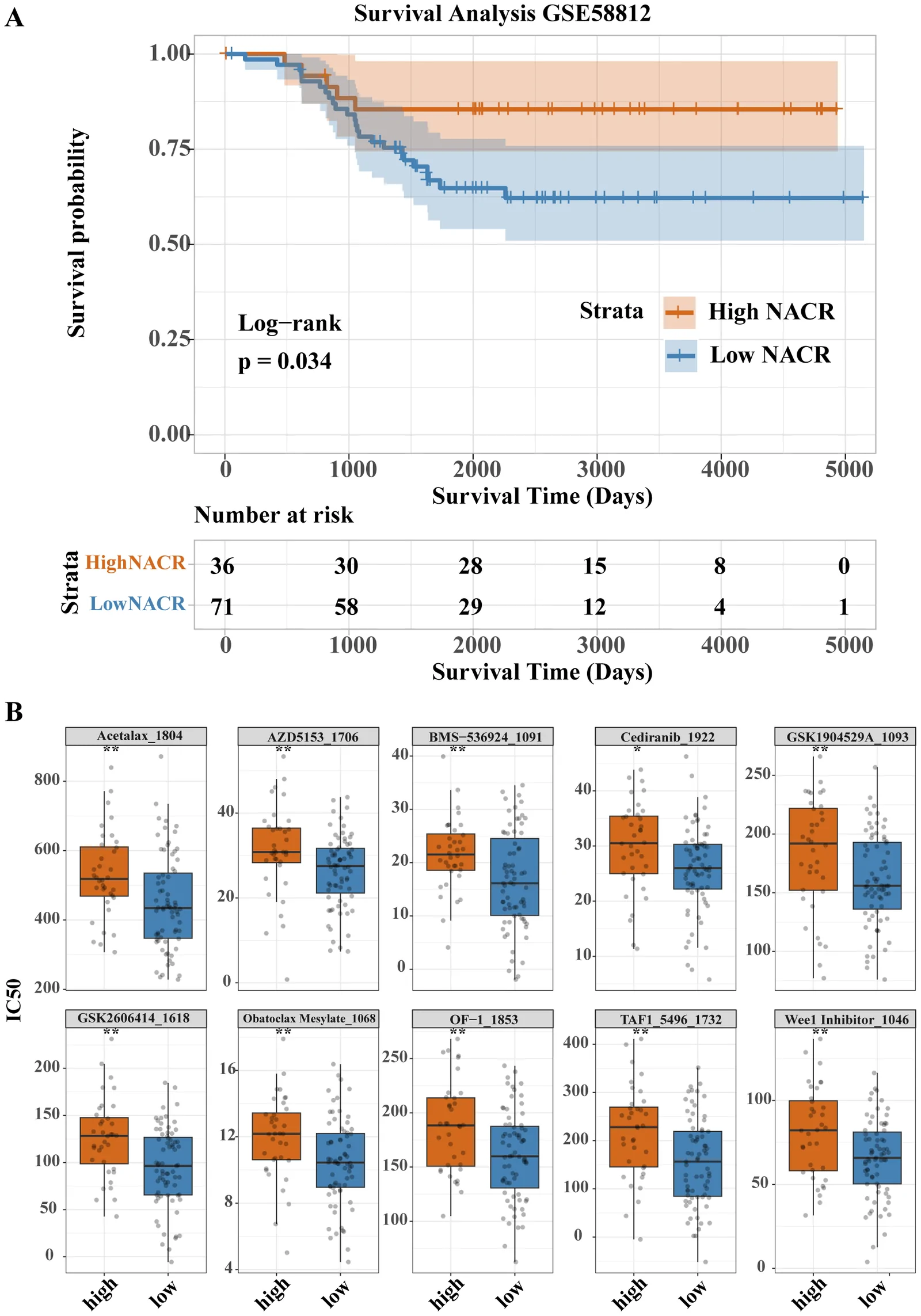
Prognostic value of the NACR signature and prediction of potential therapeutic agents. **(A)** Kaplan-Meier overall survival (OS) analysis for patients in the GSE58812 dataset stratified by high and low neoadjuvant chemotherapy response (NACR) scores (log-rank test, p = 0.034). The table below indicates the number of patients at risk at different time points. **(B)** Boxplots showing the estimated half-maximal inhibitory concentration (IC50) values of representative small molecule drugs (Acetalax, AZD5153, BMS-536924, Cediranib, GSK1904529A, GSK2606414, Obatoclax Mesylate, OF-1, TAF1_5496, and Wee1 Inhibitor) between the predicted NACR-high and predicted NACR-low groups. The boxes represent the interquartile range (IQR), and the horizontal lines indicate the medians. Statistical significance was determined using the Wilcoxon rank-sum test (*p < 0.05, **p < 0.01).

We next investigated the clinical relevance of the neoadjuvant chemotherapy response (NACR) signature. Survival analysis using the GSE58812 dataset demonstrated that patients in the NACR score-defined predicted NACR-high group had significantly better overall survival than those in the NACR score-defined predicted NACR-low group (log-rank p = 0.034; **Figure 8A**), indicating that high NACR potential is associated with favorable prognosis. Given the varied chemosensitivity within the TME, we further sought to identify alternative therapeutic agents for patients with different NACR states. We predicted the IC50 values of various small-molecule inhibitors. The results revealed that the predicted NACR-low group (which exhibited lower odds of response to standard chemotherapy) displayed significantly lower estimated IC50 values for specific targeted drugs, including Acetalax, AZD5153, BMS-536924, Cediranib, GSK1904529A, GSK2606414, Obatoclax Mesylate, OF-1, TAF1_5496, and Wee1 Inhibitor (**Figure 8B**). These findings suggest that patients with low NACR scores, who are likely resistant to conventional neoadjuvant chemotherapy, might be more sensitive to these agents, highlighting potential alternative therapeutic strategies.

### 3.8 Experimental validation of drug sensitivity and marker genes in C2 and C4 subtypes

**Figure 9:**
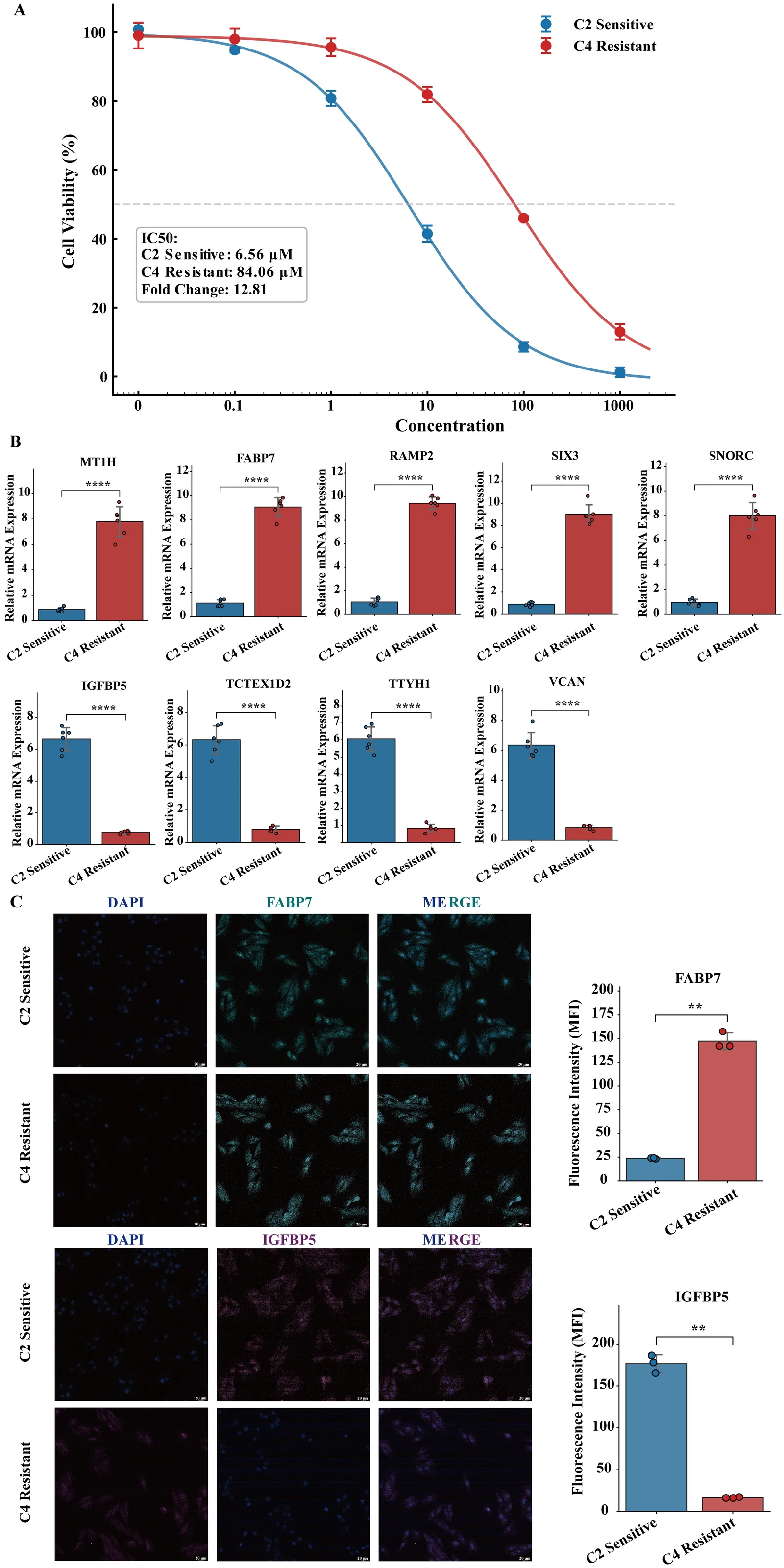
Experimental validation of differential chemotherapy sensitivity and marker gene expression in MDA-MB-231-derived C2-like and C4-like subtypes. **(A)** Dose-response curves of cell viability in representative C2-like sensitive and C4-like resistant TNBC cell models treated with varying drug concentrations. The calculated IC50 values and fold change are indicated (IC50: C2-like Sensitive = 6.56 μM, C4-like Resistant = 84.06 μM; Fold Change = 12.81; n = 6 biological replicates per group). **(B)** Bar graphs showing the relative mRNA expression levels of specific marker genes (MT1H, FABP7, RAMP2, SIX3, SNORC, IGFBP5, TCTEX1D2, TTYH1, and VCAN) in the C2-like sensitive and C4-like resistant cells, as determined by RT-qPCR. Data are presented as mean ± SD from six biological replicates per group (n = 6). ****p < 0.0001. **(C)** Representative immunofluorescence (IF) images (left) and corresponding quantification of mean fluorescence intensity (MFI) (right) for FABP7 and IGFBP5 proteins in the C2-like sensitive and C4-like resistant cells. Nuclei were counterstained with DAPI (blue). Scale bar = 20 μm. Data are presented as mean ± SD. **p < 0.01.

To experimentally validate our scRNA-seq findings, we assessed the drug sensitivity of representative MDA-MB-231-derived C2-like sensitive and C4-like resistant TNBC cell models. Cell viability assays (n = 6 biological replicates per group) demonstrated that the C2-like sensitive group was profoundly more susceptible to chemotherapy, exhibiting an IC50 of 6.56 μM compared to 84.06 μM for the C4-like resistant group (Fold Change: 12.81; **Figure 9A**). This supports the bioinformatics predictions that the C2-like subtype corresponds to high chemosensitivity, whereas C4 is associated with chemoresistance. We further validated the subtype-specific molecular signatures using RT-qPCR (n = 6 biological replicates per group). Consistent with the transcriptomic data, the C4-like resistant cells displayed significantly higher mRNA expression of MT1H, FABP7, RAMP2, SIX3, and SNORC compared to the C2-like sensitive group (**Figure 9B**). Conversely, the expression levels of IGFBP5, TCTEX1D2, TTYH1, and VCAN were markedly elevated in the C2-like sensitive cells (**Figure 9B**). To corroborate these findings at the protein level, immunofluorescence staining was performed. FABP7 exhibited strongly enriched fluorescence intensity in the C4-like resistant cells, whereas IGFBP5 protein was highly expressed in the C2-like sensitive cells (**Figure 9C**). Collectively, these experimental results support the molecular characteristics and differential chemotherapy response phenotypes of the distinct TNBC cell subtypes.

## 4 Discussion

Our single-cell RNA-seq (scRNA-seq) analysis of triple-negative breast cancer (TNBC) unveils a complex landscape of cellular heterogeneity and dynamic microenvironmental interactions that underpin variable responses to neoadjuvant chemotherapy (NAC). TNBC is distinguished by profound intratumoral heterogeneity, aggressive clinical behavior, and higher relapse rates compared to other breast cancer subtypes (23). While NAC represents the standard of care aimed at achieving a pathological complete response (pCR)—which is strongly prognostic for improved long-term survival (24)—a substantial cohort of patients exhibits *de novo* or acquired chemoresistance. Recent high-resolution transcriptomic studies highlight that distinct malignant epithelial states dictate trajectory outcomes during neoadjuvant treatments (25). Consistent with this, our sub-clustering analysis identified four distinct cancer cell subtypes (C1-C4) with divergent NAC response (NACR) profiles. We demonstrate that the C2-like subtype is significantly enriched in predicted NACR-high groups. This chemo-susceptibility may be linked to specific differentiation transcriptional programs, contrasting with the C3 and C4-like subtypes, which are associated with low-NACR, chemoresistant phenotypes. Notably, recent spatial and single-cell investigations have underscored that therapy-resistant TNBC cells often adopt partial epithelial-to-mesenchymal transition (EMT) or basal-epithelial states to evade cytotoxicity (26). While C4 was enriched in apoptosis-related pathways, its low NACR scores suggest that apoptotic gene networks in this subtype might reflect pro-survival or stress-adaptive regulatory programs rather than genuine susceptibility to drug-induced apoptosis, a phenomenon increasingly recognized in refractory TNBC populations (27).

The tumor microenvironment (TME) serves as a critical determinant of immune evasion and therapeutic efficacy in TNBC (28). Our analysis of tumor-associated macrophages (TAMs) identified three functional subsets with divergent prognostic implications for chemotherapy sensitivity (29). Specifically, TAMs + SLPI were enriched in predicted NACR-high groups, whereas TAMs + CXCL9 trended toward predicted low-response profiles. Recent single-cell and spatial mapping efforts have similarly demonstrated the extreme spatial heterogeneity of TAMs within the breast cancer microenvironment, identifying highly immunosuppressive clusters (such as SPP1+ or M2-like TAMs) that co-localize with fibroblasts to actively exclude T cells and promote resistance (30). Our findings corroborate the emerging paradigm that TAM polarization is not binary, but rather exists as a spectrum of functional states that critically shape the efficacy of cytotoxic chemotherapy, underscoring the necessity of evaluating immune cell functional heterogeneity when predicting NAC outcomes (31).

Integrated CytoTRACE 2 and Monocle 2 pseudotime analyses provided a temporal framework to evaluate developmental plasticity. We observed a striking correlation between the developmental progression of cancer cells (from the least differentiated C4 to the more differentiated C2) and monotonically increasing NACR scores. This indicates that transcriptomic maturity and differentiation status at the single-cell level profoundly impact therapeutic vulnerability, a concept supported by recent studies exploring how transcriptomic diversity marks developmental potential and chemoresistance (32). Conversely, TAMs exhibited decreasing NACR scores along their developmental trajectories, suggesting that terminally differentiated TAM subsets might orchestrate a microenvironmental remodeling phase that fosters drug resistance. Mapping these complex developmental trajectories is essential to decode the stage-specific gene regulatory networks dictating treatment failure (33).

Furthermore, intercellular communication networks unveiled distinct interaction paradigms segregating high and low NACR tumors. High-NACR ecosystems were characterized by robust communication between cancer-associated fibroblasts (CAFs) and cancer cells, which could potentially enhance drug penetrance or sensitize tumors via specific extracellular matrix (ECM) remodeling. In stark contrast, low-NACR (chemoresistant) tumors were dominated by endothelial cell–CAF and endothelial cell–TAM interactions, primarily driven by the PDGFB-PDGFRB and ICAM2-ITGAM/ITGB2 signaling axes. PDGF signaling is known to promote CAF activation and pathological angiogenesis, while ICAM2-ITGAM-mediated interactions can facilitate immune cell exclusion (34). Recent high-impact spatial transcriptomics studies have confirmed that ECM-remodeling CAFs directly interact with immunosuppressive TAMs to form fibrotic, therapy-resistant niches within TNBC (35). The specific signaling hubs we identified mirror these structural barriers and provide a potential rationale for stromal-associated chemotherapy resistance.

The translational clinical relevance of our scRNA-seq-derived signature was substantiated using an independent TNBC cohort (GSE58812), wherein higher NACR scores were associated with improved overall survival (log-rank p = 0.034). Acknowledging the urgent need for alternative strategies in refractory disease, our drug sensitivity predictions identified several candidate agents—such as Acetalax, AZD5153, BMS-536924, Cediranib, and Wee1 Inhibitor—that demonstrated lower estimated IC50 values specifically in the predicted NACR-low group. We subsequently validated these computational predictions *in vitro*, confirming a profound 12.81-fold difference in chemosensitivity between representative MDA-MB-231-derived C2-like and C4-like models. Furthermore, subtype-specific marker expression (including MT1H, FABP7, RAMP2, SIX3, SNORC in C4; and IGFBP5, TCTEX1D2, TTYH1, VCAN in C2) was supported at both the mRNA and protein levels. In conclusion, our comprehensive single-cell analysis identifies the C2-like cancer subtype and TAMs + SLPI as positive prognostic indicators, while demarcating C3/C4-like, TAMs + CXCL9, and PDGFB-PDGFRB signaling interactions as candidate resistance-associated features. These insights offer a candidate molecular framework and potential therapeutic targets to advance precision oncology for TNBC.

Several limitations should be considered. First, the NACR score represents a transcriptomic prediction rather than direct pathological response for samples without matched NAC outcome data. Second, CellChat infers ligand-receptor communication from expression data and does not prove functional signaling. Third, *in vitro* validation was performed using MDA-MB-231-derived C2-like and C4-like models, and additional TNBC cell lines, patient-derived models, and perturbation experiments will be needed to confirm the generalizability and causal roles of the identified markers and signaling axes. Finally, potential batch effects and cohort-specific biases may remain despite normalization and cross-dataset validation.

## 5 Conclusion

In this study, we leveraged scRNA-seq to dissect the cellular heterogeneity and TME dynamics underlying variable NAC responses in TNBC. Our analyses identified chemotherapy-responsive cancer cell (C2) and TAM (TAMs + SLPI) subtypes, revealed opposing NACR score trajectories during cancer cell and TAM differentiation through integrated CytoTRACE 2 and pseudotime analyses, and uncovered distinct cell-cell communication networks differentiating predicted NACR-high from predicted NACR-low tumors. These findings provide candidate biomarkers (C2, TAMs + SLPI) and actionable therapeutic targets (e.g., PDGFB-PDGFRB axis) for precision TNBC therapy. Future studies incorporating larger patient cohorts, independent validation of the NACR gene set, and functional experiments will be essential to translate these single-cell insights into clinical practice.

## Data Availability

The raw data supporting the conclusions of this article will be made available by the authors, without undue reservation.

## Supplementary material

Supplementary Table 1 NACR gene set used for scoring.

Supplementary Table 2 RT-qPCR primer sequences used for marker-gene validation.

## References

[1] Zagami P Carey LA . Triple negative breast cancer: Pitfalls and progress. NPJ Breast Cancer. (2022) 8:95. doi: 10.1038/s41523-022-00468-0 35987766 PMC9392735

[2] Yin L Duan JJ Bian XW Yu SC . Triple-negative breast cancer molecular subtyping and treatment progress. Breast Cancer Res. (2020) 22:61. doi: 10.1186/s13058-020-01296-5 32517735 PMC7285581

[3] Marra A Trapani D Viale G Criscitiello C Curigliano G . Practical classification of triple-negative breast cancer: intratumoral heterogeneity, mechanisms of drug resistance, and novel therapies. NPJ Breast Cancer. (2020) 6:54. doi: 10.1038/s41523-020-00197-2 33088912 PMC7568552

[4] van den Ende NS Nguyen AH Jager A Kok M Debets R van Deurzen CHM . Triple-negative breast cancer and predictive markers of response to neoadjuvant chemotherapy: A systematic review. Int J Mol Sci. (2023) 24:2969. doi: 10.3390/ijms24032969 36769287 PMC9918290

[5] Antonini M Mattar A Pereira TM Oliveira LL Teixeira MD Amorim AG . Pathologic complete response and breast cancer survival post-neoadjuvant chemotherapy: A systematic review and meta-analysis of real-world data. Heliyon. (2025) 11:e43069. doi: 10.1016/j.heliyon.2025.e43069 38826717

[6] Vuletic A Mirjacic Martinovic K Jurisic V . The role of tumor microenvironment in triple-negative breast cancer and its therapeutic targeting. Cells. (2025) 14:1353. doi: 10.3390/cells14171353 40940764 PMC12428307

[7] Martorana TM Ricciardi G Tralongo P Fiorentino V Pizzimenti C FranChina M . Targeting the tumor microenvironment in triple-negative breast cancer: Biological insights and therapeutic opportunities. Pathol Res Pract. (2025) 275:156226. doi: 10.1016/j.prp.2025.156226 40957300

[8] Zhang Y Wang D Peng M Tang L Ouyang J Xiong F . Single-cell RNA sequencing in cancer research. J Exp Clin Cancer Res. (2021) 40:81. doi: 10.1186/s13046-021-01874-1 33648534 PMC7919320

[9] Ren X Zhang L Zhang Y Li Z Siemers N Zhang Z . Insights gained from single-cell analysis of immune cells in the tumor microenvironment. Annu Rev Immunol. (2021) 39:583–609. doi: 10.1146/annurev-immunol-110519-071134 33637019

[10] Xin Y Ma Q Deng Q Wang T Wang D Wang G . Analysis of single-cell and spatial transcriptomics in TNBC cell-cell interactions. Front Immunol. (2025) 16:1521388. doi: 10.3389/fimmu.2025.1521388 40079015 PMC11897037

[11] Wang X Venet D Lifrange F Larsimont D Rediti M Stenbeck L . Spatial transcriptomics reveals substantial heterogeneity in triple-negative breast cancer with potential clinical implications. Nat Commun. (2024) 15:10232. doi: 10.1038/s41467-024-54145-w 39592577 PMC11599601

[12] Edgar R Domrachev M Lash AE . Gene Expression Omnibus: NCBI gene expression and hybridization array data repository. Nucleic Acids Res. (2002) 30:207–10. doi: 10.1093/nar/30.1.207 11752295 PMC99122

[13] Satija R Farrell JA Gennert D Schier AF Regev A . Spatial reconstruction of single-cell gene expression data. Nat Biotechnol. (2015) 33:495–502. doi: 10.1038/nbt.3192 25867923 PMC4430369

[14] Hafemeister C Satija R . Normalization and variance stabilization of single-cell RNA-seq data using regularized negative binomial regression. Genome Biol. (2019) 20:296. doi: 10.1186/s13059-019-1874-1 31870423 PMC6927181

[15] van der Maaten L Hinton G . Visualizing data using t-SNE. J Mach Learn Res. (2008) 9:2579–605. doi: 10.1093/oso/9780199546329.003.0006

[16] Benjamini Y Hochberg Y . Controlling the false discovery rate: A practical and powerful approach to multiple testing. J R Stat Soc Ser B Stat Methodol. (1995) 57:289–300. doi: 10.1111/j.2517-6161.1995.tb02031.x 40046247

[17] Gulati GS Sikandar SS Wesche DJ Manjunath A Bharadwaj A Berger MJ . Single-cell transcriptional diversity is a hallmark of developmental potential. Science. (2020) 367:405–11. doi: 10.1126/science.aax0249 31974247 PMC7694873

[18] Qiu X Mao Q Tang Y Wang L Chawla R Pliner HA . Reversed graph embedding resolves complex single-cell trajectories. Nat Methods. (2017) 14:979–82. doi: 10.1038/nmeth.4402 28825705 PMC5764547

[19] Gu Z Eils R Schlesner M . Complex heatmaps reveal patterns and correlations in multidimensional genomic data. Bioinformatics. (2016) 32:2847–9. doi: 10.1093/bioinformatics/btw313 27207943

[20] Jin S Guerrero-Juarez CF Zhang L Chang I Ramos R Kuan CH . Inference and analysis of cell-cell communication using CellChat. Nat Commun. (2021) 12:1088. doi: 10.1038/s41467-021-21246-9 33597522 PMC7889871

[21] Geeleher P Cox NJ Huang RS . pRRophetic: An R package for prediction of clinical chemotherapeutic response from tumor gene expression levels. PloS One. (2014) 9:e107468. doi: 10.1371/journal.pone.0107468 25229481 PMC4167990

[22] Yang W Soares J Greninger P Edelman EJ Lightfoot H Forbes S . Genomics of Drug Sensitivity in Cancer (GDSC): A resource for therapeutic biomarker discovery in cancer cells. Nucleic Acids Res. (2013) 41:D955–61. doi: 10.1093/nar/gks1111 23180760 PMC3531057

[23] Karaayvaz M Cristea S Gillespie SM Patel AP Mylvaganam R Luo CC . Unravelling subclonal heterogeneity and aggressive disease states in TNBC through single-cell RNA-seq. Nat Commun. (2018) 9:3588. doi: 10.1038/s41467-018-06052-0 30181541 PMC6123496

[24] Ahmadimoghari F Zhang Y Mavrommatis I Thakur S Starling C Roxanis I . Abstract B059: Epigenetically defined sub-clonal heterogeneity drives therapy resistance in triple-negative breast cancer. Cancer Res. (2024) 84 (3_Supplement_1):B059. doi: 10.1158/1538-7445.ADVBC23-B059 36230740

[25] Park I Shin SJ Go H Ko J Bae SJ Ahn SG . Spatial analysis of tumor immune microenvironment of TNBC with different neoadjuvant chemotherapy outcomes using multiplex immunofluorescence. Breast Cancer Res. (2025) 27:181. doi: 10.1186/s13058-025-02132-4 41121362 PMC12539054

[26] Wang Y Harada-Shoji N Kitamura N Yamazaki Y Ebata A Amari M . Mitochondrial dynamics as a novel treatment strategy for triple-negative breast cancer. Cancer Med. (2024) 13:e6987. doi: 10.1002/cam4.6987 38334464 PMC10854452

[27] So JY Ohm J Lipkowitz S . Triple negative breast cancer (TNBC): Non-genetic tumor heterogeneity and immune microenvironment: Emerging treatment options. Pharmacol Ther. (2022) 237:108253. doi: 10.1016/j.pharmthera.2022.108253 35872332 PMC9378710

[28] Xiao Y Ma D Zhao S Suo C Shi J Xue M . Multi-omics profiling reveals distinct microenvironment characterization and suggests immune escape mechanisms of triple-negative breast cancer. Clin Cancer Res. (2019) 25:5002–14. doi: 10.1158/1078-0432.CCR-18-3524 30837276

[29] Tang Y Xu A Xu Z Xie J Huang W Zhang L . Multi-omics analyses of the heterogenous immune microenvironment in triple-negative breast cancer implicate UQCRFS1 potentiates tumor progression. Exp Hematol Oncol. (2025) 14:85. doi: 10.1186/s40164-025-00672-1 40524231 PMC12172350

[30] Ravi K Zhang Y Sakala L Manoharan TJM Pockaj B LaBaer J . Tumor microenvironment on-a-chip and single-cell analysis reveal synergistic stromal-immune crosstalk on breast cancer progression. Adv Sci (Weinh). (2025) 12:2413457. doi: 10.1002/advs.202413457 40056038 PMC12021108

[31] Zhou L Yu CW . Epigenetic modulations in triple-negative breast cancer: Therapeutic implications for tumor microenvironment. Pharmacol Res. (2024) 204:107205. doi: 10.1016/j.phrs.2024.107205 38719195

[32] Ji S Yu H Zhou D Fan X Duan Y Tan Y . Cancer stem cell-derived CHI3L1 activates the MAF/CTLA4 signaling pathway to promote immune escape in triple-negative breast cancer. J Transl Med. (2023) 21:721. doi: 10.1186/s12967-023-04532-6 37838657 PMC10576881

[33] Wu Y Yi Z Li J Wei Y Feng R Liu J . FGFR blockade boosts T cell infiltration into triple-negative breast cancer by regulating cancer-associated fibroblasts. Theranostics. (2022) 12:4564–80. doi: 10.7150/thno.68972 35832090 PMC9254240

[34] Kwa MJ Adams S . Checkpoint inhibitors in triple-negative breast cancer (TNBC): Where to go from here. Cancer. (2018) 124:2086–103. doi: 10.1002/cncr.31272 29424936

[35] Sabit H Amro A Abdelfattah MM Ramadan RM Nazih M Abdel-Ghany S . The role of tumor microenvironment and immune cell crosstalk in triple-negative breast cancer (TNBC): Emerging therapeutic opportunities. Cancer Lett. (2025) 628:217865. doi: 10.1016/j.canlet.2025.217865 40516902

